# Incisional Hernia Following Open Pancreaticoduodenectomy: Incidence and Risk Factors at a Tertiary Care Centre

**DOI:** 10.3390/curroncol30080514

**Published:** 2023-07-25

**Authors:** Carolina González-Abós, Catalina Pineda, Carlos Arrocha, Jordi Farguell, Ignacio Gil, Fabio Ausania

**Affiliations:** 1 Department of HBP and Transplant Surgery, Hospital Clínic, University of Barcelona, 08036 Barcelona, Spainjfarguell@clinic.cat (J.F.); igill@clinic.cat (I.G.); ausania@clinic.cat (F.A.); 2 Gene Therapy and Cancer, Institut d’Investigacions Biomediques August Pi i Sunyer (IDIBAPS), 08036 Barcelona, Spain

**Keywords:** incisional hernia, pancreaticoduodenectomy, incidence

## Abstract

(1) Background: Incisional hernia (IH) is one of the most common complications following open abdominal surgery. There is scarce evidence on its real incidence following pancreatic surgery. The purpose of this study is to evaluate the incidence and the risk factors associated with IH development in patients undergoing pancreaticoduodenectomy (PD). (2) Methods: We retrospectively reviewed all patients undergoing PD between 2014 and 2020 at our centre. Data were extracted from a prospectively held database, including perioperative and long-term factors. We performed univariate and multivariate analysis to detect those factors potentially associated with IH development. (3) Results: The incidence of IH was 8.8% (19/213 patients). Median age was 67 (33–85) years. BMI was 24.9 (14–41) and 184 patients (86.4%) underwent PD for malignant disease. Median follow-up was 23 (6–111) months. Median time to IH development was 31 (13–89) months. Six (31.5%) patients required surgical repair. Following univariate and multivariate analysis, preoperative hypoalbuminemia (OR 3.4, 95% CI 1.24–9.16, *p* = 0.01) and BMI ≥ 30 kg/m^2^ (OR 2.6, 95% CI 1.06–8.14, *p* = 0.049) were the only factors independently associated with the development of IH. (4) Conclusions: The incidence of IH following PD was 8.8% in a tertiary care center. Preoperative hypoalbuminemia and obesity are independently associated with IH occurrence following PD.

## 1. Introduction

Pancreaticoduodenectomy (PD) is a complex procedure and it is the treatment of choice for pancreatic head and periampullary tumours. However, it is associated with high morbidity and mortality, and, despite the standardisation of the PD surgical technique and advancement of postoperative management, the morbidity and mortality rate remains high, up to 40–50% and 2–5%, respectively [1]. There is increasing evidence that this operation can be safely performed by minimally invasive surgery in selected cases. Recently, minimally invasive PD has been evaluated in large studies, and several randomised clinical trials (RCTs) have been conducted to analyse the role of laparoscopy and robotics. Two randomised clinical trials published in 2017 and 2018 comparing open vs. laparoscopic PD showed a shorter hospital stay in the laparoscopy group and comparable morbidity and mortality between the two groups. However, in 2019, the results showed by the LEOPARD 2 study raised serious concerns about laparoscopic PD [2]. This study was prematurely stopped due to a high procedure-related mortality in the laparoscopic group when compared to open surgery (10% vs. 2%). The spread of robotic surgery seems to increase the feasibility of performing minimally invasive PD; however, no randomised trials are available.

Pancreatic surgery is demanding due to its retroperitoneal location, anatomical complexity, and proximity to major vessels, and PD is still widely performed by open surgery, accessing the abdomen usually either by a bilateral subcostal or by a midline incision [1].

Whilst perioperative complications such as postoperative pancreatic fistula (POPF), delayed gastric emptying (DGE), and postoperative haemorrage have been widely studied and some mitigation strategies have been published [3,4], there are very little data about the incidence and risk factors associated with incisional hernia (IH) development in PD patients.

The occurrence of IH is one of the most common complications of open abdominal surgical procedures. Previous authors demonstrated that the incidence of IH following laparotomy ranges between 5 and 25%, and it can increase up to 40% in high-risk patients [5,6]. Several factors have been associated with IH development, such as the male gender, age (>55 years), type of incision (midline laparotomy and subcostal incision with midline extension), surgical site infection, obesity, smoking habit, and preoperative chemotherapy [7]. Comorbidities such as diabetes mellitus (DM) and chronic obstructive pulmonary disease (COPD) can also increase the risk of developing IH [5,8,9,10,11].

The clinical presentation of IH can range from asymptomatic cases detected incidentally during routine imaging to severe cases presenting with symptoms such as pain, discomfort, and the presence of a palpable mass. The consequences of an IH can have a long-term impact on patients’ life, leading to impaired quality of life, reduced physical function, and potential requirement of additional surgical interventions [12]. IH can also require emergency surgery for small bowel incarceration [2,5]. However, there are no studies evaluating possible factors associated with IH development in PD patients; therefore, no prophylaxis policy can be advised in high-risk patients.

The management of incidental hernias after open pancreaticoduodenectomy can be challenging due to the poor oncological outcomes of the patients. The treatment approach depends on various factors, including the size and location of the hernia, the presence of symptoms, and the overall health status of the patient. The surgical repair may involve a variety of techniques, including open or laparoscopic approaches, with the goal of reinforcing the weakened abdominal wall and reducing the risk of recurrence.

The aim of the current study is to evaluate the incidence of IH and identify possible risk factors associated with IH development in a single-centre cohort of consecutive patients who underwent PD.

## 2. Materials and Methods

We performed a retrospective review of patients who underwent a PD between 2014 and 2020 at Hospital Clinic, Barcelona, Spain. Data were extracted from a prospectively held database. Missing data for the purpose of the study was retrospectively collected. Patients’ characteristics and clinical data were reviewed to determine potential factors associated with IH development, such as demographic data and preoperative comorbidities that could affect wound healing.

All patients that receive pancreatic surgery were routinely followed-up in a surgical outpatient clinic on a 6-months basis by also performing a CT scan and tumour markers. Additionally, patients were also followed-up by the oncology department especially during the first 6 months if they received adjuvant treatment. Those patients receiving surgery for benign diseases were usually followed up in the outpatient clinic by MRI rather than CT scan.

### 2.1. Definition of IH

Incisional hernia is a discontinuity in the abdominal fascia, shown as a protrusion of the intraperitoneal structures through the defect of the abdominal wall at the incision site [13]. The presence of clinical or radiological (CT) evidence of IH at the time of outpatient follow-up was recorded.

### 2.2. Surgical Procedure

All patients were operated on in the same hospital and under the same conditions. PDs were performed by senior surgeons with extensive surgical experience in hepatobiliary and pancreatic (HPB) surgery. Antibiotic prophylaxis (piperacillin + tazobactam) was routinely administered at anesthetic induction and repeated in case of surgery duration > 6 h or blood loss > 400 mL as previously described [14]. Postoperative recommendations of short period of physical rest and a postoperative binder was uniformly advised to all patients.

Two types of incisions were used:

(1) Bilateral subcostal incision, defined as the incision horizontal to the costal margin, beginning below the xiphoid and extending laterally;
(2) J-shaped incision, defined as a transverse incision in the right hypochondrium with vertical extension in the midline to the xiphoid.

For fascia closure, a double layer of continuous polydioxanone suture (PDS loop n. 1) was used, following the rule of 4:1 [15]. As it is recommended, the use of a continuous-suture technique using a monofilament suture material tied with self-locking knots, avoid excessive tension placed on the suture.

In case of postoperative wound dehiscence, full-thickness abdominal wall closure was performed. Sutures are placed at a fairly large distance from the wound edge, and often a distance of 3 cm is necessary. The stitch should include all layers of the abdominal wall except the skin, as a classic mass-closure stitch. Placing stitches at an interval of 4 to 5 mm means that the tension is distributed on a large volume of tissue, decreasing the risk of the suture cutting through the tissues [15]. Abdominal drains were placed in all patients to obtain correct postoperative control.

Patients undergoing minimally invasive pancreatic resection were excluded.

Postoperative patient care was performed according to the protocol of the centre with serial blood tests with C-reactive protein and testing of drain amylase levels on the 3rd and 5th postoperative days. All patients were advised to avoid lifting weights and were recommended to wear an abdominal girdle while standing and walking during the first 30 days after surgery.

### 2.3. Data Extraction

The data were extracted from a prospectively held database and double-checked with medical records during follow-up. Demographic variables (age, gender, weight, height, and body mass index) and associated comorbidities (hypertension, diabetes mellitus, chronic obstructive pulmonary disease, and associated malignancy) were included.

American Society of Anesthesiologists score (ASA score), preoperative albumin, and preoperative renal function were also collected. Obesity was defined according to the World Health Organization as BMI ≥ 30 kg/m^2^ [16,17].

Intraoperative data, such as need for blood products, type of surgical incision, technique of reconstruction, and the suture used for fascia closure, were recorded. Postoperative complications, need for reoperation, and readmission were included in the analysis.

Clinically relevant postoperative pancreatic fistula, postoperative haemorrhage, and delayed gastric emptying were defined according to the International Study Group on Pancreatic Fistula (ISGPS) definitions [18,19,20].

Surgical site infection (SSI) was defined as an infection related to a surgical procedure that occurs near the surgical site within 30 days following surgery (or up to 90 days following surgery where an implant is involved). SSIs were classified as superficial (involving only skin and subcutaneous tissues), deep (softer tissues), and organ or space (include abscess or anastomotic leak) [20].

The follow-up period was considered until the last outpatient clinic or death.

### 2.4. Statistical Analysis

The statistical analyses were performed with the Statistical Package for the Social Sciences (SPSS Statistics version 25, IBM, Chicago, IL, USA). For two-sided group comparisons, the chi-square test or Fisher’s exact test were used for categorical variables and the Mann–Whitney U test was applied for continuous variables. Binary and linear regression analyses were performed to control for the effects of covariates on the clinical outcomes by including in the multivariate analysis all the variables that reached *p* values < 0.05 in the univariate analyses. Receiver operating characteristic (ROC) curves were constructed to determine cut-off values by the Youden index. A *p* value < 0.05 was considered statistically significant.

## 3. Results

During the study period, a total of 270 patients underwent PD in our centre and 213 patients met the inclusion criteria. Seven patients undergoing minimally invasive PD were excluded. Thirty (12.3%) patients were excluded for the following intraoperative findings: 22 patients were considered unresectable at a laparotomy for a locally advanced tumour (12 cases), liver metastases (5 cases), and peritoneal metastases (5 cases); 6 patients eventually required a total pancreatectomy, and 2 patients underwent an extended pancreaticoduodenectomy. Patients’ characteristics are shown in Table 1. IH occurred in 19 (8.8%) patients

Median age was 67 (33–85) years, and median BMI was 24.9 kg/m^2^ (14–41). Out of 213, 184 (86.4%) patients underwent surgery for malignant disease. Subcostal bilateral incision was performed in 187 (87.8%) cases and J-shaped incision in 26 (12.2%) patients. Clavien–Dindo ≥ 3 complications occurred in 37 (17.4%) patients. CR-POPF occurred in 49 (23%) patients and DGE in 40 (18.8%). Reoperation was indicated in 28 (13.1%) cases and abdominal wound dehiscence occurred in 5 (2.3%) cases. Superficial, deep, and space surgical site infections occurred in 4 (1.9%), 5 (2.3%), and 44 (20.7%) cases.

IH occurred in 19 (8.8%) patients. Median diameter of IH was 7.2 cm (3–28 cm) according to CT findings. Median time to IH occurrence was 31 (13–89) months. Of the six (31.5%) patients who were submitted to hernia repair, two of them were as emergency surgery for incarcerated hernia. The remaining 13 patients were not submitted to surgery for the following reasons: cancer-related death (*n* = 3), poor performance status (*n* = 4), and tumour relapse (*n* = 6). Ten patients with IH showed a significantly lower preoperative hypoalbuminemia (<3.4 g/dL).

In univariate analysis, preoperative hypoalbuminemia (OR 0.93, 95% CI 0.87–0.98, *p* = 0.02), BMI ≥ 30 kg/m^2^ (OR 1.13, 95% CI 1.03–1.25, *p* = 0.01), and abdominal wall dehiscence (OR 0.13, 95% CI 0.02–0.85, *p* = 0.03) were significantly associated with IH occurrence.

In multivariate analysis, preoperative hypoalbuminemia (OR 3.41, 95% CI 1.24–9.37, *p* = 0.02) and BMI ≥ 30 kg/m^2^ (OR 2.6, 95% CI 1.06–8.14, *p* = 0.049) were independently associated with IH development (Table 2).

## 4. Discussion

In this study, we found that IH occurs in 8.8% of patients undergoing PD. Multivariate analysis revealed that preoperative hypoalbuminemia and obesity are independently associated with IH development.

Incisional hernia is a frequent complication of abdominal surgery. Occurrence of IH depends on several preoperative factors, such as older age, smoking, male gender, obesity, previous laparotomy, diabetes, COPD, and presence of malignancy [21,22]. Intraoperative factors, such as suture closure technique (suture length/wound length ratio 4:1) and polydioxanone suture have also been recognised as protective factors [23].

In major abdominal surgery, a previously published Cochrane systematic review and a randomised double-blind equivalence trial suggested that there is no statistically significant difference in IH rates according to the type of incision [24,25]. Finally, wound complications and mechanical stress may play an important role in the development of late IH. Coughing, abdominal distension, heavy physical exercise, straining during defecation, or vomiting after operation may increase the risk of IH independently of any other associated factors [22,23].

Pancreaticoduodenectomy is a complex procedure used to treat benign and malignant periampullary lesions. Life expectancy is increasing due to recent improvements in early diagnosis, and an increased surgical approach due to neoadjuvant treatment. Evidence suggests that the 5-year survival for resected pancreatic adenocarcinoma (PDA) has incrementally improved over time as a result of more effective adjuvant treatment, use of neoadjuvant therapy, and an increase in margin negative resections over the last two decades [2,26]. Thus, the population of long-term survivors following PD is increasing, developing late complications of the procedure such as incisional hernias.

IH in PD patients has not been previously evaluated in detail. Chen-Xu et al. previously found that the incidence of incisional hernia following pancreatic surgery can reach 11.6% of patients; however, in this study, all patients undergoing any type of pancreatic resection were included and the real incidence of IH in PD patients is unclear. [8] A recent meta-analysis which focused on IH following hepatobiliary and pancreatic surgery showed that there is limited evidence to guide the incision choice and methods of closure in HBP surgery [27]. However, we think PD patients represent a specific population with a particular risk of complications, and, therefore, they should not be mixed with other types of patients at the time of statistical analysis.

In our study, an albumin level < 3.4 gr/dL, BMI ≥ 30 kg/m^2^, and abdominal wall dehiscence were significant risk factors for incisional hernia formation following PD, and the outcome of incisional hernia was not influenced by the incision performed and suturing technique.

Both factors are strongly related to sarcopenia and they are well-known factors associated with complications in abdominal and pancreatic surgery [28,29].

There are several mechanisms that contribute to the development of hypoalbuminemia in pancreatic cancer patients. The tumour itself can lead to malnutrition and weight loss, known as cancer cachexia. Pancreatic cancer disrupts the normal metabolic processes, leading to decreased appetite, impaired digestion and absorption of nutrients, and increased energy expenditure. This chronic state of malnutrition contributes to a decline in albumin production, leading to hypoalbuminemia [30]. Additionally, head pancreatic cancer usually obstructs the bile duct, impairing the flow of bile from the liver to the bowel. This obstruction hinders the absorption of fat-soluble vitamins, such as vitamin D, which is necessary for the synthesis of albumin [28,29]. The reduced availability of vitamin D further exacerbates hypoalbuminemia in pancreatic cancer patients.

Regarding high BMI, there has been a significant rise in the incidence of obesity worldwide over the past few decades,. It is associated with chronic low-grade inflammation: adipose tissue releases inflammatory molecules known as adipokines, which can contribute to the development of a proinflammatory state. Persistent inflammation can damage DNA, disrupt normal cellular processes, and increase the risk of cancer initiation and progression. It is also well-known that obesity also has an impact on cancer outcomes and patient prognosis. Furthermore, obesity is associated with increased difficulty in pancreatic surgery, due to a more challenging anatomical recognition and surgical manipulation. A fatty pancreas is also associated with an increased risk of postoperative pancreatic fistula. Finally, the increased surgical difficulty in obese patients also extends to postoperative care and recovery. Obese individuals are at a higher risk of developing surgical site infections, wound healing complications, and deep vein thrombosis due to impaired blood flow and delayed wound healing.

Our findings are in line with previous literature, suggesting that the obesity/low muscle mass combination, appropriately defined as sarcopenic obesity, is associated with poorer postoperative outcomes even in ventral hernia repair [31]. However, due to the retrospective nature of the study, we were unable to calculate the body composition in these patients.

Prevention of IH development in these patients is challenging because this is a multifactorial complication. Some patient risk factors such as nutritional status and BMI can sometimes be modified before surgery through prehabilitation programs [32]. However, these options are not always feasible [33].

Several studies have shown the benefits of the use of prophylactic mesh in high-risk patients to reduce the formation of incisional hernias after major surgery. These studies have consistently demonstrated a significant reduction in the incidence of incisional hernias compared to conventional closure techniques [34]. In addition to lowering hernia rates, prophylactic mesh has also shown benefits in terms of reducing pain, improving quality of life, and decreasing the need for subsequent surgeries [11,12,35]. However, their acceptance has not been widely adopted, presumably due to mesh-related problems such as mesh infection and chronic pain [36,37,38]. In our study, the SSI rate was 24.9% and reoperation rate was 13.8%, indicating that the use of mesh could increase the severity of postoperative complications in a significant number of patients, and, therefore, the optimization of preoperative factors seems a wiser recommendation in this setting. Ongoing research is focused on optimising prophylactic mesh placement by refining mesh materials, mesh fixation methods, and patient selection criteria. Additionally, efforts are underway to better identify high-risk patients who would most benefit from prophylactic mesh placement. Long-term follow-up studies are needed to assess the durability and cost-effectiveness of prophylactic mesh in reducing the incidence of incisional hernias and associated complications.

The spread of MIS could lead to a decrease of IH incidence, but it is not well-defined. MIS has not been well-standardised for PD patients, but robotic surgery and centralisation in expert centres will increase the adoption of MIS PD. Robotic-assisted pancreaticoduodenectomy offers several potential benefits compared to the laparoscopic approach [2]. The use of robotic instruments with articulating arms and 3D visualisation provides surgeons with enhanced dexterity and depth perception, enabling them to navigate the challenging anatomy of the pancreas and surrounding structures more accurately. Moreover, robotic technology allows for better access to difficult-to-reach areas, enabling surgeons to perform more precise dissections and reconstructions. Minimally invasive surgery with smaller incisions can minimise postoperative pain and reduce the risk of complications. It is important to note that, while minimally invasive surgery generally shows a lower incidence of incisional hernias, the risk may still exist, especially in certain patients or complex procedures.

The present study had some limitations. First, the retrospective nature of the study could have led to poor control over the exposure factor, covariates, and potential confounders. However, data were extracted from a prospectively held database. Second, only patients from a single institution were considered, and, therefore, our results might not be reproduced in other centres; however, homogeneity of surgical procedures and postoperative management increases the clinical significance of our results. Also, the short median follow-up associated with the poor prognosis of most patients might have underestimated the long-term incidence and complications of IH [39]. The number of patients included in this study is not very high: however, data collection before the study time was not performed on a prospectively held database and, therefore, we preferred to avoid a potential quality bias. Finally, some potential factors associated with IH development were not considered for this study: (1) postoperative ileus was not included because it can be difficult to differentiate it from delayed gastric emptying or other complications related to CR-POPF; (2) malnutrition was not described as we did not routinely perform a preoperative objective test on these patients; however, we did use hypoalbuminemia as a surrogate; and (3) the incidence of previous abdominal open surgery was very low (<4%) in our cohort and, therefore, was not statistically relevant.

In conclusion, we demonstrated that the incidence of incisional hernia in open PD patients is 8.8%, and that lower albumin levels and obesity are significant risk factors associated with IH development, while the type of incision was not associated with an increased rate of IH. Preoperative identification of high-risk patients could be useful to assess the impact of preoperative and intraoperative pre-emptive measures. However, these factors are difficult to optimise in patients with pancreatic cancer because this particular disease and the need for urgent treatment make preoperative optimisation difficult.

In the case of other indications with a better prognosis, prehabilitation could represent a promising intervention to minimise postoperative morbidity and speed recovery. However, patients’ response to preoperative optimisation is unpredictable, and there are no studies confirming the real benefit in pancreatic surgery. Further studies should assess the impact of prehabilitation in pancreatic cancer patients and the outcomes in IH development. Nevertheless, the identification of factors associated with IH development could be also very useful for detecting patients who are better candidates for a remote follow-up at some stage [40].

## Figures and Tables

**Table 1:** Demographic, clinical, and perioperative variables.

Variable	Total (*n* = 213)	IH (*n* = 19)	Absence of IH	*p* Value
			(*n* = 194)	
**Male gender (%)**	**126 (59.2)**	**11 (57.9)**	115 (59.3)	0.91
Median age, years (range)	67.08 (33–85)	69.58 (48–81)	66.83 (33–85)	0.28
Median BMI, kg/m^2^ (range)	24.96 (14–41)	27.36 (22–37)	24.72 (14–41)	0.01
BMI ≥ 30 kg/m^2^ (%)	19 (8.9%)	5 (35%)	14 (7)	0.005
Malignancy (%)	184 (86.4)	16 (84.2)	168 (86.6)	0.77
CR-POPF (%)	49 (23)	4 (21.1)	45 (23.2)	1
Comorbidity (%)				
Diabetes	39 (18.3)	4 (21.1)	35 (18)	0.75
COPD	11 (5.2)	1 (5.3)	10 (5.2)	1
Hypertension	105 (49.3)	13 (68.4)	92 (47.4)	0.08
Smoker	75 (35.2)	5 (26.3)	70 (36.1)	0.46
ASA III-IV (%)	62 (29.1)	4 (21.1)	58 (29.9)	0.59
Preoperative hypoalbuminemia, <3.4 g/dL (%)	54 (25.4)	10 (52.6)	44 (22.7)	0.004
Chronic renal failure (%)	12 (5.6)	1 (5.3)	11 (5.7)	1
Bilateral subcostal incision (%)	187 (87.8%)	15 (78.9%)	172 (88.7%)	0.26
Reoperation (%)	28 (13.1%)	3 (15.8%)	25 (12.9%)	0.72
Abdominal wall dehiscence (%)	5 (2.3%)	2 (10.5%)	3 (1.5%)	0.06
Blood transfusion (%)	17 (8%)	3 (15.8%)	14 (7.2%)	0.77
Blood loss > 700 mL (%)	22 (10.3%)	3 (15.8%)	19 (9.8%)	0.42
Surgical site infection (%)	53 (24.9%)	2 (10.5%)	51 (26.3%)	0.17
Organ space SSI	44 (20.7%)	1 (5.3%)	43 (22.2%)	0.08

BMI: body mass index, COPD: chronic obstructive pulmonary disease, ASA: American Society of Anesthesiologists score; SSI: surgical site infection.

**Table 2:** Variables affecting likelihood to develop postoperative incisional hernia after pancreatoduodenectomy in univariable and multivariable analysis.

Variable	Univariate Analysis		Multivariate Analysis	
	Odds Ratio (95% IC)	*p* Value	Odds Ratio (95% IC)	*p* Value
Gender	0.94 (0.36–2.45)	0.9	-	-
Age	1.02 (0.97–1.07)	0.34	-	-
**BMI ≥ 30 kg/m^2^**	**1.13 (1.03–1.25)**	**0.01**	**2.601 (1.06–8.14)**	**0.049**
**Preoperative** **hypoalbuminemia**	**0.93 (0.87–0.98)**	**0.02**	**3.418 (1.24–9.16)**	**0.01**
ERF	0.92 (0.11–7.57)	0.94	-	-
Malignancy	1.21 (0.33–4.44)	0.77	-	-
Diabetes	0.83 (0.26–2.65)	0.75	-	-
COPD	0.98 (0.11–8.12)	0.98	-	-
Smoker	1.58 (0.54–4.57)	0.39	-	-
ASA III-IV	1.59 (0.50–5.02)	0.42	-	-
CR-POPF	0.80 (0.30–2.14)	0.72	-	-
Type of incision	2.08 (0.63–6.84)	0.22	-	-
Blood loss > 700 mL	1.72 (0.46–6.47)	0.42	-	-
Blood transfusion	0.41 (0.10–1.59)	0.2	-	-
Surgical site infection	3.11 (0.69–13.93)	0.13	-	-
**Abdominal wall dehiscence**	**0.13 (0.02–0.85)**	**0.03**	5.53 (0.74–41.3)	0.09
Reoperation	0.78 (0.21–2.90)	0.72	-	-

BMI: body mass index, COPD: chronic obstructive pulmonary disease, ASA: score American Society of Anesthesiologists score.

## Abbreviations

IH	incisional hernia
PD	pancreaticoduodenectomy
BMI	body mass index
POPF	postoperative pancreatic fistula
DGE	delayed gastric emptying
DM	diabetes mellitus
COPD	chronic obstructive pulmonary disease
CT	computed tomography
MRI	magnetic resonance imaging
HPB	hepatobiliary and pancreatic
PDS	polydioxanone
ASA	American Society of Anesthesiologists score
HT	hypertension
ISGP	International Study Group on Pancreatic Fistula
SSI	surgical site infection
ROC	receiver operating characteristic
CR-POPF	clinically relevant postoperative pancreatic fistula
ERF	chronic renal failure
PDA	pancreatic adenocarcinoma
